# Supplementary material for: Benchmarking tomographic acquisition schemes for high-resolution structural biology
Source: Nat Commun. 2020 Feb 13;11:876. doi: 10.1038/s41467-020-14535-2 (PMC7018747; doi:10.1038/s41467-020-14535-2)
Supplement: Supplementary file 1 — Supplementary Information [file 41467_2020_14535_MOESM1_ESM.pdf]

Supplementary Information

Benchmarking Tomographic Acquisition Schemes for High-Resolution Structural Biology

Turoňová et al.

Supplementary Note 1: Bidirectional scheme starting at -20°

An additional dataset using bidirectional scheme with an offset, starting with [-20°:61°] followed by [-23°:-59°], was acquired to test its potential for high-resolution SA. Since the acquisition was done on a different grid than for the other datasets, an additional control dataset was collected using the dose-symmetric scheme (on the same grid). The acquisition parameters remained the same.

The results are summarized in Supplementary figure 3. The resolution obtained with the control dataset was 4.2 Å, and thus same as for the original dose-symmetric dataset, while the bidirectional scheme starting at -20° resulted in a structure with resolution of 4.7 Å – thus the numerical improvement over the bidirectional scheme starting at 0° is marginal (~0.1 Å). The B-factor analysis further underscores that the differences between both bidirectional schemes are minor. The analysis of structural features, however, shows that the averages from the new datasets are not as well resolved as the ones from the original grid (see the Supplementary Figure 3).

Supplementary Note 2: Correction for local distortions

The correction for local distortions was performed on the dose-symmetric and the bidirectional dataset using the method described in [1], adapted to fit into our processing workflow. The final averages together with their positions and orientations were used to create new fiducials. A new alignment was computed in eTomo using local alignments. Since novaCTF does not currently support the local alignments, this analysis was done using tomograms corrected with 2D-CTF strip-based approach from IMOD (ctfphaseflip). The positions and orientations from the final alignments were used to directly compute the final average from 2D-CTF corrected tomograms, and lead to worse resolution as compared to 3D-CTF correction, as expected. A new set of 2D-CTF corrected tomograms was reconstructed using the local refinements, followed by SA. The obtained nominal resolution was the same as without the local alignments for the dose-symmetric scheme and improved only marginally for the bidirectional scheme (see Supplementary Figure 4).

We thus conclude that correcting for local distortions does not have a significantly different impact on the bidirectional scheme than on the dose-symmetric.

Supplementary Note 3: Particle cleaning

The particle cleaning was done fully automatically after 2 iterations of alignment with 8x binned data. Two different cleaning methods were used. (i) Geometric-based which discards misaligned particles and (ii) distance-based which removes the initial oversampling. For geometric cleaning an ellipsoid was fitted into each VLP and the angular distance between an ideal and actual orientation of each particle as well as its distance from the fitted ellipsoid (i.e. its residual) is computed. Particles with angular distance and/or residual larger than the respective standard deviation were discarded. The geometric-based cleaning does not require any user-specified parameter and provides useful statistics on the VLPs quality. The distance-based cleaning discards particles that shifted to the same position during the alignment. The distance is a user-specified parameter and corresponds to the physical distance among the hexamers in the lattice. The criterion for choosing the better particle was angular distance. As shown in Supplementary Figure 5, the particle cleaning worked reasonably well for both full and incomplete VLPs. Roughly 30% of particles were discarded by the geometric-based cleaning and around 60% by the distance-based cleaning (see the Supplementary Table 3).

The distance-based cleaning was also performed after the processing of 4x and 2x binned data to further discard the particles that moved to the same positions – the criterion for choosing the better particle was constrained cross-correlation (CC) value from the last alignment. The number of particles discarded during this cleaning was for both cases negligible.

At last, a particle cleaning was performed prior the alignment of unbinned data. A normal distribution was fitted into the mean density values of subtomograms and subtomograms with mean value less or greater than the standard deviation were discarded.

Supplementary Table 1: Overview of software and parameters used for processing.

Step	Software	Parameters	
Image acquisition	SerialEM 3.7.0. beta build 1/11/19<br><br>GMS 3.32.1508.0		
CTF Estimation	CTFFind4 v.4.1.8	Pixel size<br>Acceleration voltage<br>Spherical aberration<br>Amplitude contrast<br>Power spectrum size<br>Minimum resolution<br>Maximum resolution<br>Minimum defocus<br>Maximum defocus<br>Defocus step<br>Astigmatism<br><br>For DS VPP def:<br>Minimum phase-shift<br>Maximum phase-shift<br>Phase-shift search step	1.3269 Å<br>300 keV<br>2.7 mm<br>0.07<br>512 pixels<br>30 Å<br>5 Å<br>10000 Å<br>75000 Å<br>500 Å<br>100<br><br><br>1.22 rad<br>2.1 rad<br>0.1 rad
Dose-exposure correction	Matlab script from [2]		
High-peaks removal	eTomo v.4.9.2 (ccderaser)	Peak criterion<br>Difference criterion<br>Maximum radius<br>Extra-large difference criterion	10<br>8<br>4.2<br>19
Cross-correlation alignment	eTomo (tiltxcorr)	Default parameters	
Fiducial model generation	eTomo using "Make seed and track" option<br><br>Seed model (autofidseed)<br><br>Track beads (beadtrack)	 <br><br>Default parameters<br><br>Sobel filter<br>Fill seed model gaps<br>Local tracking<br>Local area size	 <br><br><br><br>1.5 – 3<br>True<br>True<br>1000
Alignment transformation computation	eTomo (tiltalign)	Do not sort fiducial into 2 surfaces<br>Rotation solution type<br>Magnification solution type<br>Tilt angle solution type<br>Distortion solution type<br>Beam tilt	True<br>No rotation<br>Fixed at 1.0<br>Fixed<br>Disabled<br>No
Preliminary 8x binned reconstruction	eTomo (tilt)	Logarithm of densities<br>Radial filtering cutoff<br>Radial filtering falloff<br>SIRT-like filter	No<br>0.35<br>0.035<br>15 iterations
Reconstruction	novaCTF	Correction type<br>Astigmatism correction	Multiplication<br>True

		Slab size	15 nm
Binning	Fourier3D		
VLPs picking	IMOD v.4.9.2		
Generation of subtomograms initial positions and orientations	Matlab script		
Subtomogram Averaging	C++ version of original TOM and AV3 scripts	See the table below	
FSC	Matlab script implementing phase-randomization as described in [3]		

**SA parameters**	**Reference**	**8x binned**	**4x binned**	**2x binned**	**Unbinned**
Box size (pixels)	36	36	72	128	192
Iterations	20	2	3	2	4
Cone angle opening (degrees)	12	48	24 12 8	8 4	4 2 2 2
Cone angle sampling	2.9	5.8	3.9 2.9 1.9	1.9 1	1
In-plane angle opening (degrees)	8	84	48 30 16	12 6	6 4 4 4
In-plane angle sampling	1	6	4 3 2	2 1	1
Low-pass filter (pixels)	12	12	12	20 31	29 32 32 32
High-pass filter (pixels)	1	1	1	1 1	1 10 10 14

Supplementary Table 2: Setup for tiltcontroller used to collect the continuous scheme.

Delay time after tilting by basic increment	10 s
Autofocus offset	0 µm
Autofocus	at least every 3 degrees
Repeat record if of field lost is more than	5%
Get tracking image when error in X/Y prediction is more than	5%
Track before autofocusing	Yes
Align with preview before getting new track reference	Yes
Get new track reference if Record alignment differs by more than	5%
Do autofocus when error in focus prediction exceeds	0.2 µm
Keep beam intensity constant	Yes
Limit image shift to	15 µm

The defocus was set manually for each tilt-series in the navigator. The delay of 10s corresponds to the typical time needed for drift stabilization during dose-symmetric acquisition and was used for the continuous scheme to ensure as close acquisition conditions as possible.

Supplementary Table 3: Overview of number of subtomograms used for initial alignment on 8x binned data and after the geometric and distance-based cleaning.

Scheme	VLPs	Initial subtomograms	After geometric-based cleaning	After distance-based cleaning	Angular distance mean	Angular distance STD
Continuous	34	220145	159767 (72.6%)	18962 (8.6%)	10.7°	15.1°
Bidirectional	57	350903	243491 (69.4%)	29791 (8.5%)	15.7°	20°
Dose-symmetric	32	199569	143571 (71.9%)	17456 (8.7%)	12.7°	18.3°
DS dec	53	329287	229834 (69.8%)	28772 (8.7%)	15.6°	20.1°
DS inc	53	306821	217922 (71%)	26404 (8.6%)	13.8°	18.9°
DS VPP foc	36	227827	157838 (69.3%)	18686 (8.2%)	17°	21.2°
DS VPP def	42	268757	188102 (70%)	22851 (8.5%)	16.4°	22.5°

The numbers correspond to the 5 tomogram subsets.

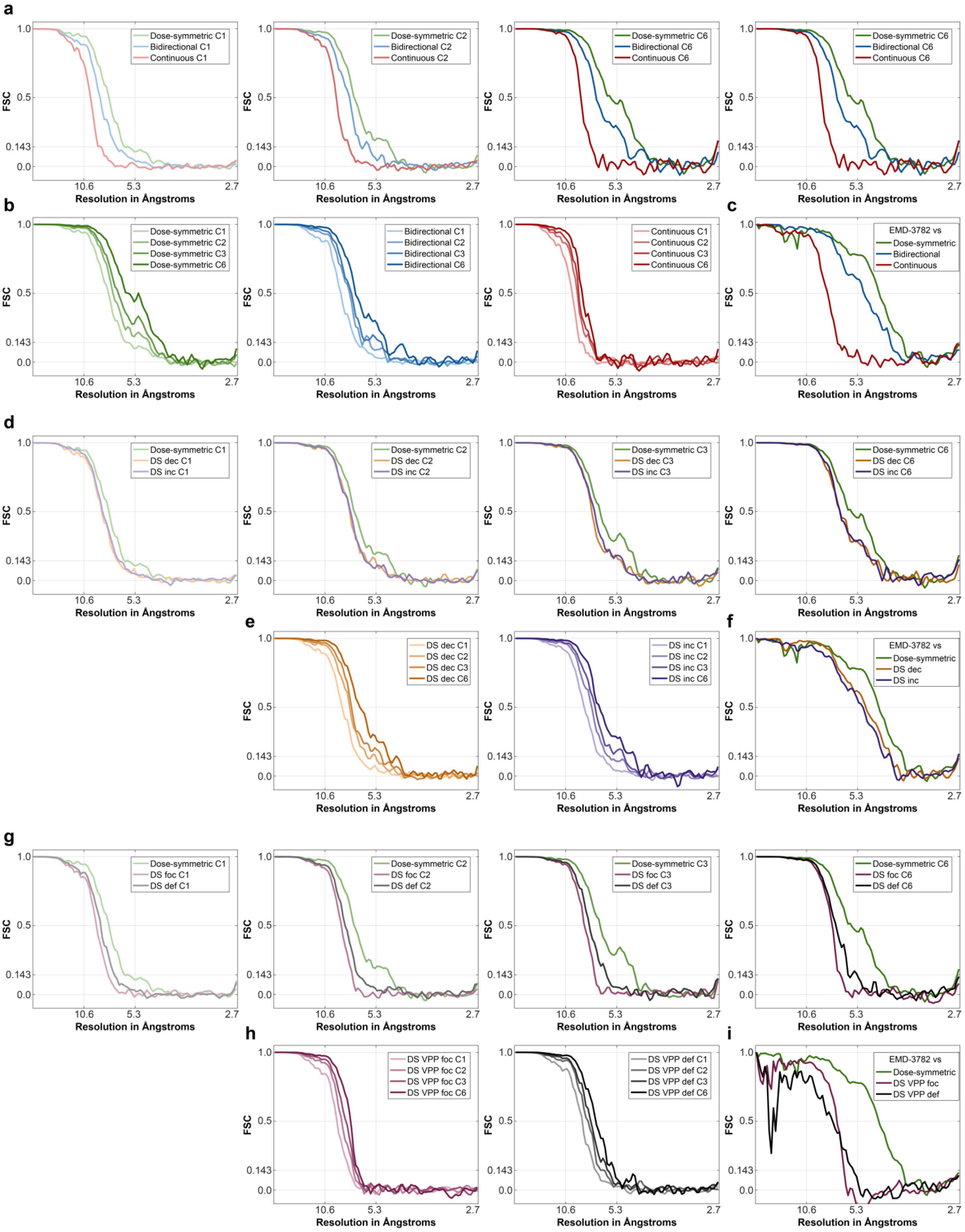
a
Dose-symmetric C1
Bidirectional C1
Continuous C1
Dose-symmetric C2
Bidirectional C2
Continuous C2
Dose-symmetric C6
Bidirectional C6
Continuous C6
b
Dose-symmetric C1
Dose-symmetric C2
Dose-symmetric C3
Dose-symmetric C6
Bidirectional C1
Bidirectional C2
Bidirectional C3
Bidirectional C6
Continuous C1
Continuous C2
Continuous C3
Continuous C6
c
EMD-3782 vs
Dose-symmetric
Bidirectional
Continuous
d
Dose-symmetric C1
DS dec C1
DS inc C1
Dose-symmetric C2
DS dec C2
DS inc C2
Dose-symmetric C3
DS dec C3
DS inc C3
Dose-symmetric C6
DS dec C6
DS inc C6
e
DS dec C1
DS dec C2
DS dec C3
DS dec C6
DS inc C1
DS inc C2
DS inc C3
DS inc C6
f
EMD-3782 vs
Dose-symmetric
DS dec
DS inc
g
Dose-symmetric C1
DS foc C1
DS def C1
Dose-symmetric C2
DS foc C2
DS def C2
Dose-symmetric C3
DS foc C3
DS def C3
Dose-symmetric C6
DS foc C6
DS def C6
h
DS VPP foc C1
DS VPP foc C2
DS VPP foc C3
DS VPP foc C6
DS VPP def C1
DS VPP def C2
DS VPP def C3
DS VPP def C6
i
EMD-3782 vs
Dose-symmetric
DS VPP foc
DS VPP def
FSC
1.0
0.5
0.143
0.0
10.6
5.3
2.7
Resolution in Angstroms

Supplementary Figure 1: FSC curves. a. FSC curves comparing the continuous, bidirectional and dose-symmetric schemes with different symmetries. **b**. Comparison of different symmetries for the schemes from A. **c.** Comparison of FSC between C6 maps from the respective schemes and the EMD-3782 map with resolution 3.9Å. **d-f**. Same as a-c, but comparing the dose-symmetric scheme with DS dec and DS inc. **g-i**. Same as a-c, but comparing the dose-symmetric scheme with DS VPP foc and DS VPP dec.

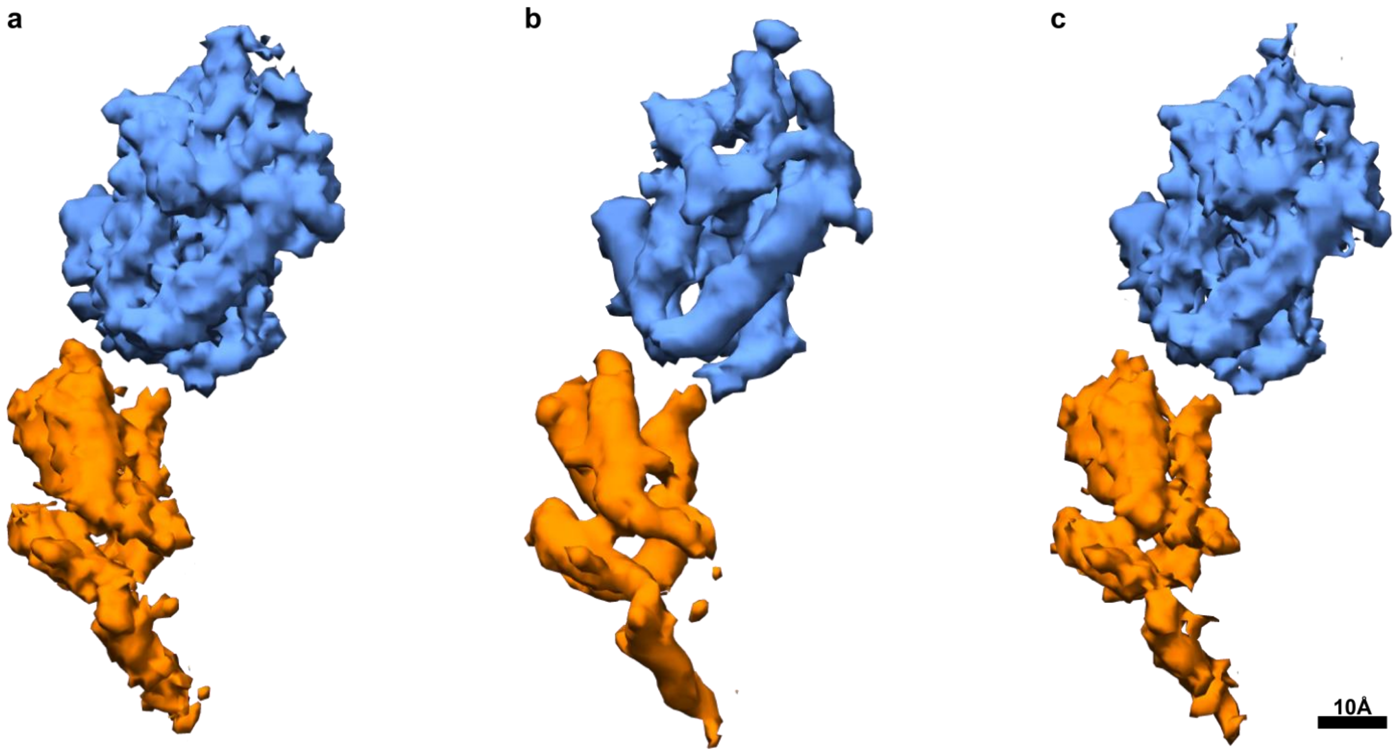

Supplementary Figure 2: Sharpening of VPP structures. a. A structure of a chain from PDB 5L93 of HIV-1 CA-SP1 monomer obtained by DS VPP foc scheme. **b.** Same as A, but sharpened using empirically determined B-factor of -600. **c.** Same as A, but filtered using the matchto filter from EMAN2 [4]. The 4.2 Å structure obtained by the dose-symmetric scheme was used as the reference for the amplitude matching.

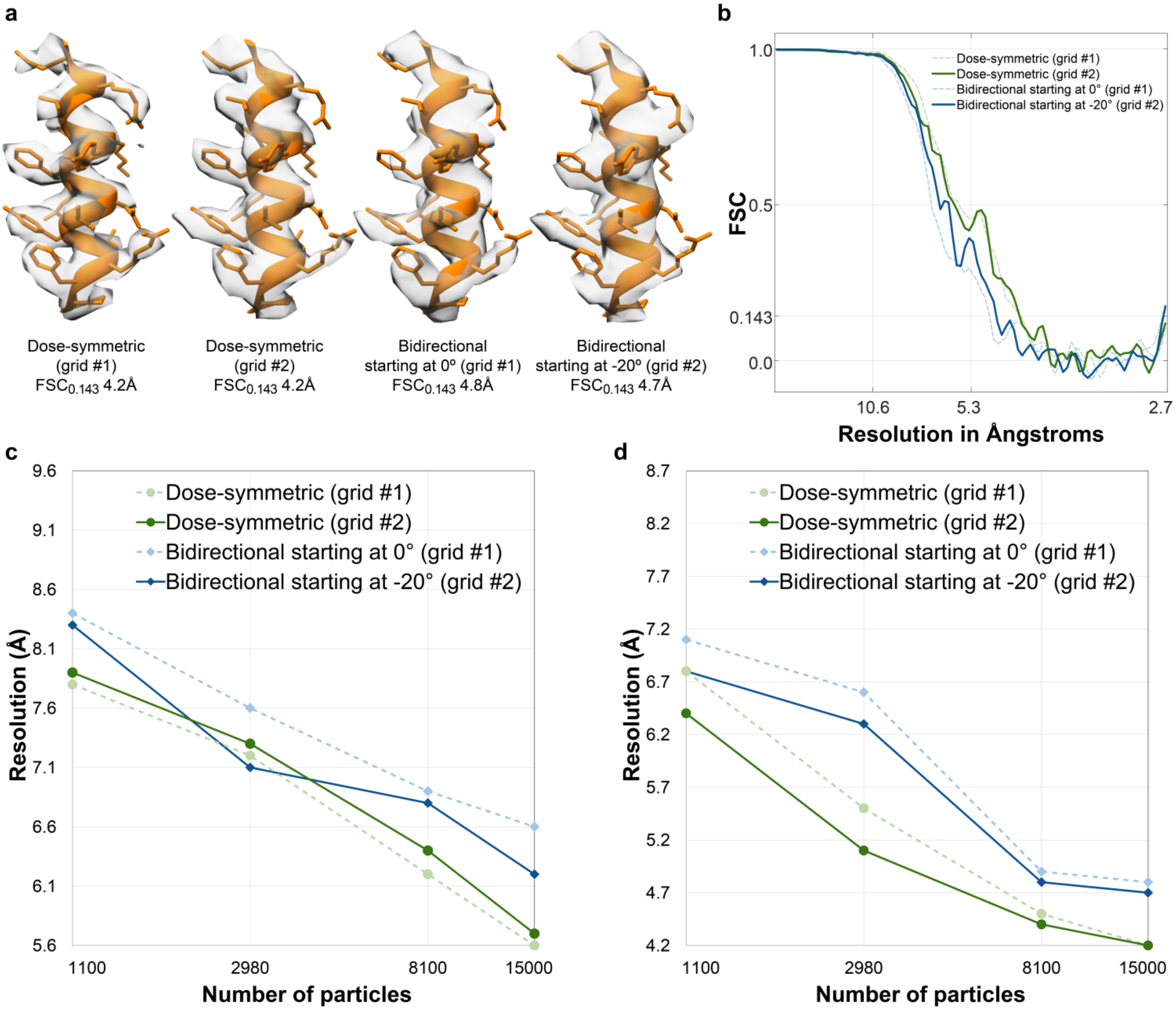

Supplementary Figure 3: Comparison of bidirectional schemes without and with a -20° offset to the dose symmetric scheme. The results are compared to the control dose-symmetric scheme collected on the same grid (#2) and to the original data collected on different grid (#1) – the bidirectional scheme starting at 0° and the dose-symmetric scheme. **a.** Structural details of individual helix of HIV-1 CA-SP1 determined by the four schemes. **b**. FSC curves. **c**. B-factor analysis. Plot of resolution of structures at 0.5 criterion as a function of number of particles (x-axis scaled logarithmically). **d.** Same as c, but the resolution was estimated at 0.143 criterion.

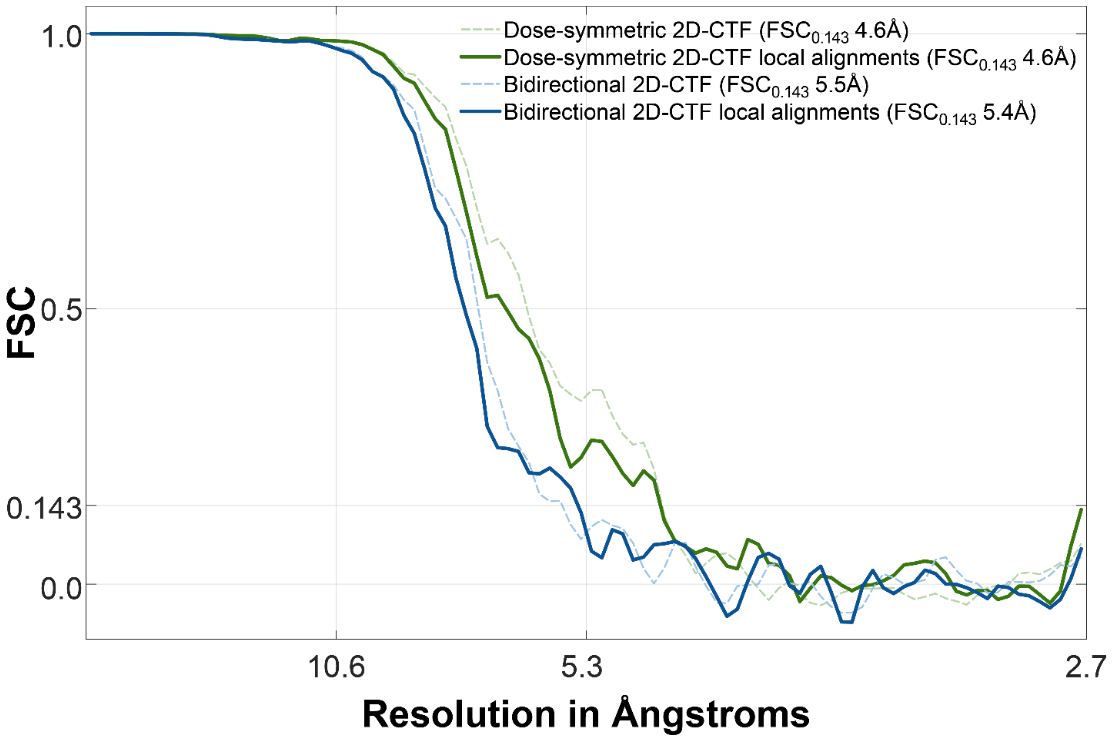

Supplementary Figure 4: Comparison of FSC of dose-symmetric and bidirectional schemes with and without correction of local distortions.

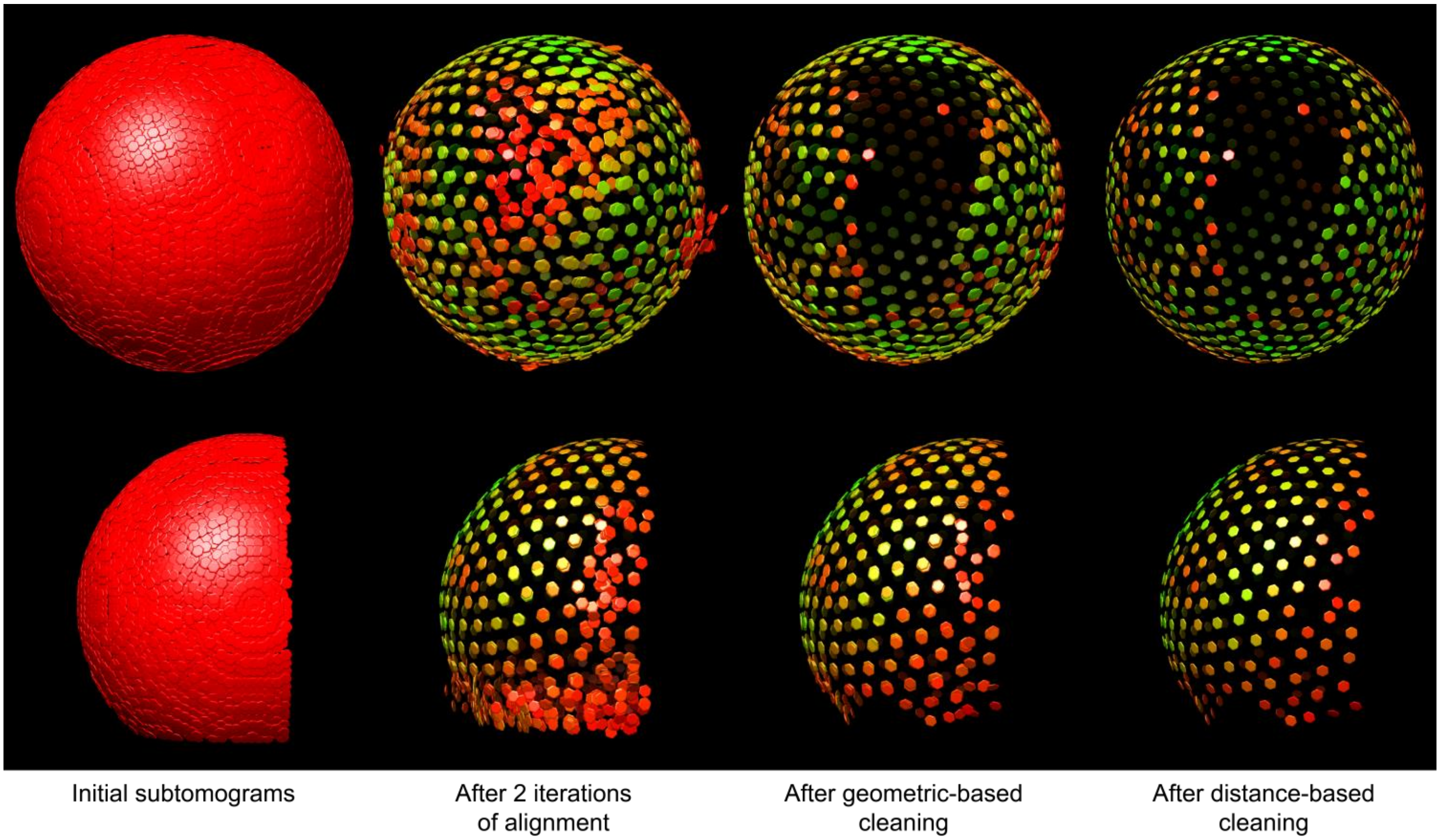

Supplementary Figure 5: Particle cleaning of VLPs. Each hexagon represents one subtomogram with red color corresponding to the lowest CC value between a subtomogram and a reference and green color corresponding to the highest CC. Initially, the VLP lattice is oversampled, i.e. on average 10x more positions are created than assumed number of subunits. After 2 iterations of alignment, the subtomograms lock to the actual lattice. The geometric-based cleaning discards the subtomograms that deviate from the lattice geometry, i.e. have large angular distance and/or large distance from an estimated VLP radius. Finally, the distance-based cleaning removes subtomograms that shifted to the same positions. Both cleaning procedures work reliably for both complete (top row) and incomplete (bottom row) VLPs. The visualization was done in Chimera [5] using Place object plug-in [6].

Supplementary References

1. Himes, B. A. & Zhang, P. emclarity: software for high-resolution cryo-electron tomography and subtomogram averaging. *Nat. Methods.* **15,** 955–961 (2018).

2. Wan, W. *et al.* Structure and assembly of the ebola virus nucleocapsid. *Nature* **551,** 394–397 (2017).

3. Chen, S. *et al.* High-resolution noise substitution to measure overfitting and validate resolution in 3d structure determination by single particle electron cryomicroscopy. *Ultramicroscopy* **135,** 24–35 (2013).

4. Tang, G. et al. Eman2: An extensible image processing suite for electron microscopy. *J. Struct. Biol.* **157**, 38–46 (2007).

5. Pettersen, E. F. et al. Ucsf chimera-a visualization system for exploratory research and analysis. *J. Comput. Chem.* **25**, 1605–1612 (2004).

6. Qu, K. et al. Structure and architecture of immature and mature murine leukemia virus capsids. *P. Natl. Acad. Sci. USA* **115**, E11751–E11760 (2018).
